# Monitoring lung injury with particle flow rate in LPS‐ and COVID‐19‐induced ARDS

**DOI:** 10.14814/phy2.14802

**Published:** 2021-07-11

**Authors:** Martin Stenlo, Iran A. N. Silva, Snejana Hyllén, Deniz A. Bölükbas, Anna Niroomand, Edgars Grins, Per Ederoth, Oskar Hallgren, Leif Pierre, Darcy E. Wagner, Sandra Lindstedt

**Affiliations:** ^1^ Department of Cardiothoracic Anaesthesia and Intensive Care and Cardiothoracic Surgery and Transplantation Skåne University Hospital Lund University Sweden; ^2^ Department of Experimental Medical Sciences Lung Bioengineering and Regeneration Lund University Sweden; ^3^ Wallenberg Center for Molecular Medicine Lund University Sweden; ^4^ Lund Stem Cell Center Lund University Sweden; ^5^ Department of Clinical Sciences Lund University Sweden; ^6^ Rutgers Robert University New Brunswick New Jersey USA

**Keywords:** acute respiratory distress syndrome, COVID‐19, extra corporal membrane oxygenation, lung injury diagnostics

## Abstract

In severe acute respiratory distress syndrome (ARDS), extracorporeal membrane oxygenation (ECMO) is a life‐prolonging treatment, especially among COVID‐19 patients. Evaluation of lung injury progression is challenging with current techniques. Diagnostic imaging or invasive diagnostics are risky given the difficulties of intra‐hospital transportation, contraindication of biopsies, and the potential for the spread of infections, such as in COVID‐19 patients. We have recently shown that particle flow rate (PFR) from exhaled breath could be a noninvasive, early detection method for ARDS during mechanical ventilation. We hypothesized that PFR could also measure the progress of lung injury during ECMO treatment. Lipopolysaccharide (LPS) was thus used to induce ARDS in pigs under mechanical ventilation. Eight were connected to ECMO, whereas seven animals were not. In addition, six animals received sham treatment with saline. Four human patients with ECMO and ARDS were also monitored. In the pigs, as lung injury ensued, the PFR dramatically increased and a particular spike followed the establishment of ECMO in the LPS‐treated animals. PFR remained elevated in all animals with no signs of lung recovery. In the human patients, in the two that recovered, PFR decreased. In the two whose lung function deteriorated while on ECMO, there was increased PFR with no sign of recovery in lung function. The present results indicate that real‐time monitoring of PFR may be a new, complementary approach in the clinic for measurement of the extent of lung injury and recovery over time in ECMO patients with ARDS.

## Graphical abstract

Particle flow rate (PFR) used in conjunction with mechanical ventilation can be used to monitor lung injury during ECMO treatment in animals with LPS induced ARDS and in humans with COVID‐19 induced ARDS. The present results indicate that real‐time monitoring of PFR may be a new, complementary approach in the clinic for measurement of the extent of lung injury and recovery over time in ECMO patients with ARDS.

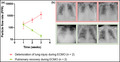

## 1 INTRODUCTION

Acute respiratory distress syndrome (ARDS) is a common cause of death with mortality rates of around 30–50% and even up to 80% in COVID‐19‐induced ARDS patients (Gonzales et al., 2015; Maca et al., 2017; Potere et al., 2020). In severe cases of ARDS, extracorporeal membrane oxygenation (ECMO) is a life‐prolonging intervention. Evaluating the status of the lung injury over time with imaging such as computer tomography (CT) is a risk. The demands of intra‐hospital transport are great, including the large number of medical staff needed and the potential of ECMO cannula dislocation, leading to complications like lethal bleeding. Invasive diagnostics, such as lung biopsies, are contraindicated for heparinized patients, which is required for ECMO (Chockalingam & Hong, 2015). In addition, in COVID‐19 patients, transport to other departments must be limited in order to protect staff and other patients from the highly contagious virus. A bedside clinical indicator to evaluate the degree of lung injury over time would be of great significant clinical importance.

We have recently shown that particle flow rate (PFR) has potential as a noninvasive tool for patients on mechanical ventilation and that PFR is elevated during the early stages of ARDS development in a porcine ARDS model (Broberg, Pierre, et al., 2019; Broberg et al., 2018, 2020; Stenlo et al., 2020). PFR is a measure of exhaled breath particles (EBPs) counted using an optical counter built into a PExA 2.0 machine customized for mechanical ventilation. EBPs are thought to originate from the respiratory tract lining fluid (RTLF) that covers the epithelial surface of the distal lung (Bake et al., 2019; Behndig et al., 2019). EBPs are transmitted in exhaled air during the opening and closing of small airways but can also be transmitted by shear stress.

The measurement of such EBPs could serve as a new clinical indicator to survey the underlying state of a patient's lungs, particularly when ECMO is used. ECMO as a supportive therapy rather than a disease‐modifying treatment may mask the true condition of the lungs, leading to a delay in the notice of clinical deterioration. We have previously shown that changes in hemodynamics can alter the PFR (9). As ECMO alters hemodynamics, we thus investigated whether PFR could reflect progressive lung injury during ECMO treatment. An LPS‐induced ARDS porcine model on ECMO as well as human patients on ECMO both with and without COVID‐19 induced ARDS was utilized in this study.

## 2 MATERIALS AND METHODS

All animals received standard care according to local and international regulations. This study was approved by the Ethics Committee (Dnr 8401/2017) for Animal Research and follows the Principles of Laboratory Animal Care of the National Society for Medical Research, USA and the Guide for the Care and Use of Laboratory Animals, published by the National Academies Press (1996).

The study on the four patients was performed in accordance with the Declaration of Helsinki and was approved by the local ethics committee (Dnr 2017/519, 2020‐01864).

### 2.1 Animal preparation

Twenty‐one pigs with a mean weight of 61.7 ± 2.2 kg were medicated and intubated according to standard techniques (see supplemental Methods). They were kept on mechanical ventilation (MV) adjusted to maintain a normal pH with a tidal volume (Vt) kept at 6–8 ml/kg.

**FIGURE 1:**
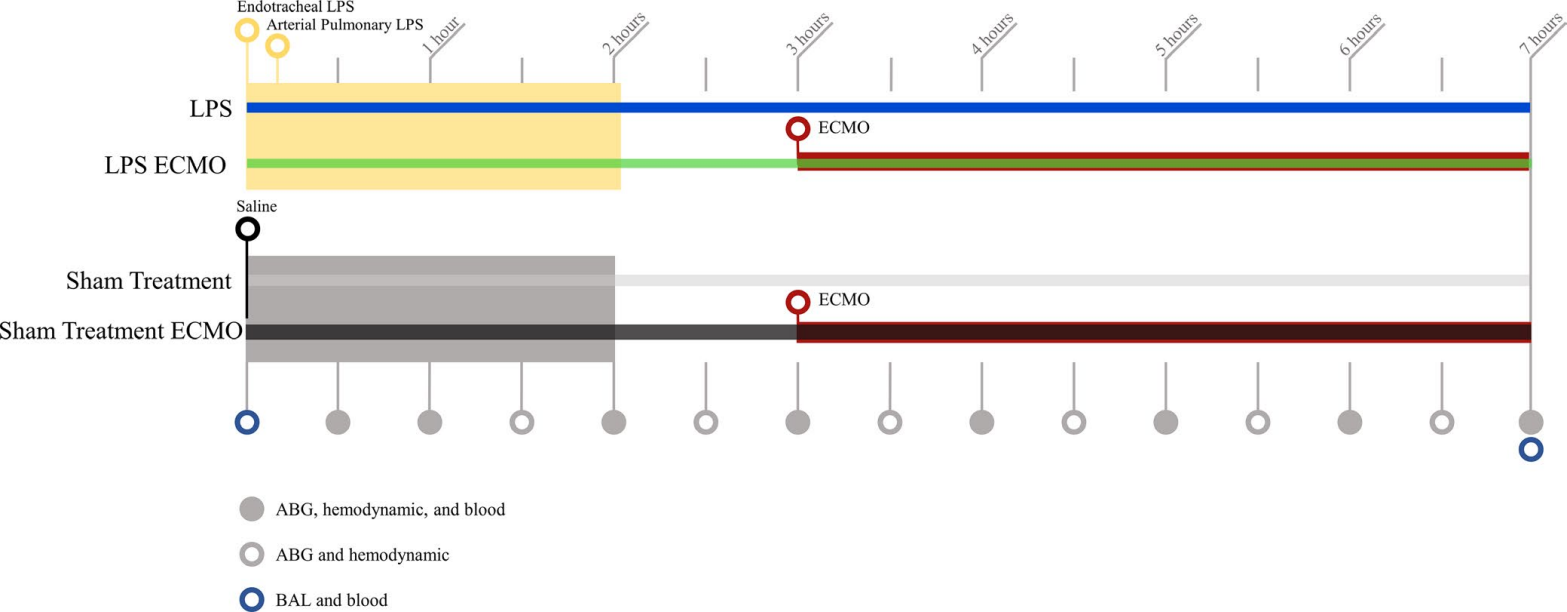
Timeline for the experimental setup. A total of 21 animals, LPS ECMO animals (*n* = 8) and sham‐treated ECMO animals (*n* = 3) received ECMO for 4 h after approximately 3 h of mechanical ventilation. In the LPS ECMO group, the ECMO was connected after ARDS was confirmed with two separate blood gases taken at 15‐min intervals. Additional seven animals that received LPS did not receive ECMO treatment (LPS animals). Finally, a sham‐treated group received only saline and mechanical ventilation referred to as sham treatment. Continuous measurements of PExA and regular hemodynamic measurements, ventilatory parameters, and blood gases were obtained. A pulmectomy was done at the end of the experiment for histological evaluation. Arterial blood gas (ABG), hemodynamic measurements, blood samples (blood), and bronchoalveolar lavage fluid (BAL) were collected at times as denoted. PFR was continuously measured throughout the duration of the experiment

Lipopolysaccharide (LPS) induced an ARDS‐like condition. Eight animals received LPS and MV followed by veno‐arterial ECMO (VA‐ECMO) for 4 h (LPS ECMO animals). Seven animals received LPS and MV (LPS animals). Three animals received saline treatment and MV for 6 h (Sham treatment animals), whereas three animals received saline treatment and MV for 3 h followed by 4 h of ECMO treatment (Sham treatment ECMO animals). The sham animal group has been previously published. The experimental timeline is shown in Figure 1.

### 2.2 Arterial blood gases

Arterial blood gases were analyzed by standard protocol every 30 min with an ABL 90 FLEX blood gas analyzer (Radiometer Medical ApS).

### 2.3 Definition of ARDS

The different ARDS stages were defined according to the Berlin criteria (Force et al., 2012).

### 2.4 Hemodynamics measurements using a Swan‐Ganz catheter and an arterial line

Hemodynamic parameters were measured every 30 min using thermodilution with a Swan‐Ganz catheter and an arterial line. The parameters are listed and reported in Tables 1 and 2.

**TABLE 1:** Physiological status of pigs treated with (a) LPS (regular) and sham (bold) over time and (b) LPS and ECMO (regular) and sham with ECMO (bold) over time

	Sat (%)	HR (bpm)	SBP (mmHg)	DBP (mmHg)	MAP (mmHg)	CVP (mmHg)	Temp (°C)
(a)							
Base	98.38 ± 0.50	101 ± 8.7	98.13 ± 4.72	71.38 ± 4.65	82.63 ± 4.84	4.25 ± 0.94	37.01 ± 0.17
	**99 ± 0.7**	**56 ± 2.8**	**96 ± 2.6**	**60 ± 2.4**	**75 ± 2.7**	**10 ± 0.7**	**37.6 ± 0.3**
30 min	96.13 ± 0.85	65.63 ± 5.5	125.5 ± 4.98	91.13 ± 4.34	108 ± 4.28	8.88 ± 1.91	36.51 ± 0.24
	**99 ± 1**	**57 ± 0.6**	**100 ± 1**	**61 ± 2.7**	**76 ± 2.7**	**10 ± 0.7**	**37.5 ± 0.4**
60 min	94.75 ± 1.08	83.13 ± 8.7	113.6 ± 5.95	79.5 ± 6.61	95.75 ± 6.54	7.63 ± 1.52	36.47 ± 0.29
	**99 ± 0.6**	**62 ± 5**	**102 ± 5.1**	**67 ± 10**	**80 ± 9.2**	**10 ± 1**	**37.5 ± 0.4**
90 min	92.5 ± 1.27	99.38 ± 3.7	94.75 ± 3.39	55.63 ± 5.04	71 ± 4.56	5.63 ± 1.55	36.3 ± 0.23
	**99 ± 0.7**	**58 ± 0.7**	**101 ± 5**	**62 ± 8.6**	**77 ± 9.5**	**10 ± 1.2**	**37.6 ± 0.5**
120 min	90.75 ± 2.13	103 ± 3.3	93.13 ± 2.74	50.75 ± 4.52	65.38 ± 4.24	6.13 ± 1.65	36.01 ± 0.27
	**99 ± 0.6**	**57 ± 0.9**	**96 ± 2.7**	**60 ± 4.7**	**75 ± 4.9**	**10 ± 1.3**	**37.5 ± 0.7**
150 min	88.57 ± 2.63	114 ± 3.5	95.29 ± 2.35	49.71 ± 4.30	64.43 ± 4.27	5 ± 0.87	35.84 ± 0.26
	**99 ± 0.3**	**58 ± 1.5**	**93 ± 1.9**	**59 ± 4.3**	**73 ± 4.2**	**9 ± 0.6**	**37.4 ± 0.8**
180 min	90 ± 8	119.5 ± 5.5	92 ± 7.0	34 ± 0	48 ± 1	4 ± 1	35.7 ± 0.1
	**99 ± 0.6**	**55 ± 2.6**	**92 ± 4**	**58 ± 5.8**	**71 ± 5.4**	**9 ± 0.3**	**37.5 ± 0.8**
b							
180 min	89.75 ± 1.93	112.3 ± 4.6	94.5 ± 2.2	47.00 ± 4.2	62.0 ± 4.3	6.4 ± 1.6	35.8 ± 0.2
	**98.67 ± 0.67**	**64 ± 6.66**	**96.3 ± 1.8**	**54 ± 4.6**	**72 ± 5.03**	**7.3 ± 2.9**	**38.2 ± 0.2**
210 min	90.63 ± 2.39	124.1 ± 6.0	85.5 ± 3.6	42.1 ± 2.4	52.4 ± 3.0	6.5 ± 1.1	35.8 ± 0.4
	**97.67 ± 1.45**	**72.3 ± 0.2**	**96.3 ± 2.0**	**64.3 ± 10.8**	**75.3 ± 7.3**	**4.7 ± 1.9**	**37.4 ± 0.2**
240 min	90 ± 3.25	135.3 ± 5.1	82.4 ± 5.4	41.6 ± 2.5	51.8 ± 2.9	7.1 ± 1.7	36.6 ± 0.3
	**97.67 ± 1.20**	**70.3 ± 3.9**	**95.7 ± 1.8**	**67 ± 11.1**	**76.7 ± 8.2**	**4.7 ± 1.9**	**36.9 ± 0.2**
270 min	89.38 ± 2.59	135.1 ± 3.4	85.3 ± 5.4	44.6 ± 3.2	55.3 ± 3.4	7.1 ± 1.6	36.8 ± 0.2
	**97.33 ± 0.88**	**79.7 ± 7.7**	**92.7 ± 2.3**	**64.3 ± 7.4**	**72.3 ± 6.8**	**4.7 ± 1.5**	**36.7 ± 0.2**
300 min	88.63 ± 3.32	149.1 ± 7.4	80.3 ± 5.3	47.9 ± 3.9	57.1 ± 4.3	7.1 ± 1.5	37.2 ± 0.3
	**96.67 ± 0.33**	**93.7 ± 5.6**	**90.7 ± 4.70**	**63.3 ± 7.4**	**71 ± 5.6**	**4.3 ± 1.8**	**36.7 ± 0.1**
330 min	86.14 ± 3.45	152 ± 6.0	84.1 ± 5.6	47.4 ± 4.6	57.1 ± 5.2	7.4 ± 1.7	37.4 ± 0.4
	**96.67 ± 0.33**	**93.7 ± 5.6**	**96 ± 7.55**	**65 ± 11.1**	**72.7 ± 10.7**	**5 ± 1.7**	**36.7 ± 0.2**
360 min	84.29 ± 2.98	154.9 ± 7.4	84 ± 5.7	47.6 ± 3.5	57 ± 4.07	7.9 ± 2.1	37 ± 0.4
	**95.67 ± 0.67**	**104.7 ± 5.6**	**97.7 ± 1.8**	**67.3 ± 7.0**	**74.3 ± 6.2**	**4.3 ± 1.8**	**36.8 ± 0.2**
390 min	80.86 ± 4.78	147.1 ± 6.9	81.1 ± 6.3	49 ± 6.1	56.7 ± 4.3	8.1 ± 1.8	36.7 ± 0.3
	**95 ± 0**	**122 ± 17.0**	**83 ± 11.0**	**52 ± 3.8**	**58.3 ± 3.7**	**4.3 ± 1.8**	**36.8 ± 0.2**
420 min	80.43 ± 5.06	152.4 ± 6.3	80.3 ± 6.0	50.1 ± 6.8	57.9 ± 6.7	8 ± 1.9	36.8 ± 0.6
	**96 ± 0.58**	**119.3 ± 19.3**	**81.7 ± 9.3**	**51.3 ± 4.7**	**57.7 ± 4.7**	**4.7 ± 1.9**	**36.7 ± 0.2**

Vitals during LPS and sham treatment and b) shows vitals during LPS ECMO treatment and sham treatment ECMO: oxygen saturation (Sat), heart rate (HR), systolic blood pressure (SBP), diastolic blood pressure (DBP), mean arterial pressure (MAP), central venous pressure (CVP), temperature (Temp).

**TABLE 2:** Physiological status of pigs treated with (a) LPS (regular) and sham (bold) over time and (b) LPS and ECMO (regular) and sham with ECMO (bold) over time

	SPP (mmHg)	DPP (mmHg)	MPP (mmHg)	Wedge (mmHg)	CO (L/min)	SVR (DS/cm^5^)	PVR (DS/cm^5^)
(a)							
Base	22 ± 1.15	12.25 ± 1.18	16.63 ± 0.80	7.25 ± 0.49	6.18 ± 0.37	1016 ± 82.6	124.1 ± 9.24
	**27 ± 1**	**14 ± 3**	**20 ± 2.5**	**15 ± 0.5**	**2.9 ± 0**	**1834 ± 136**	**113 ± 58**
30 min	48.63 ± 4.69	28.75 ± 3.03	37.88 ± 2.92	12.88 ± 1.16	3.90 ± 0.31	2202 ± 215.3	610.1 ± 84.45
	**27 ± 0**	**15 ± 2**	**20 ± 2.5**	**14 ± 0.5**	**2.9 ± 0**	**1834 ± 136**	**113 ± 58**
60 min	38.5 ± 2.31	25 ± 2.02	32.13 ± 1.61	10.13 ± 0.93	4.59 ± 0.26	1558 ± 111.7	392.4 ± 43.62
	**27 ± 0.5**	**15 ± 2.5**	**19 ± 3.5**	**14 ± 1**	**3.4 ± 0.2**	**1708 ± 144**	**154 ± 28**
90 min	33.88 ± 2.79	23.13 ± 1.95	27.88 ± 2.33	8.125 ± 0.77	5.17 ± 0.24	1008 ± 66.0	321.4 ± 43.53
	**28 ± 3**	**12 ± 1**	**18 ± 3**	**14 ± 1**	**3.4 ± 0.3**	**1666 ± 102**	**139 ± 13**
120 min	39.75 ± 2.69	24.75 ± 1.31	31.38 ± 1.40	9.25 ± 1.15	5.17 ± 0.31	929 ± 58.8	345.6 ± 34.5
	**30 ± 4**	**13 ± 2**	**19 ± 3.5**	**14 ± 0**	**3.0 ± 0.2**	**1696 ± 57**	**101 ± 73**
150 min	40.57 ± 3.58	25.57 ± 1.77	32 ± 1.60	8.43 ± 1.13	5.54 ± 0.26	857 ± 60.2	343.7 ± 31.9
	**31 ± 5**	**12 ± 1.5**	**18 ± 3**	**14 ± 0.5**	**3.0 ± 0.2**	**1696 ± 57**	**101 ± 73**
180 min	44 ± 8	31.5 ± 0.5	36 ± 2	10 ± 2	4.79 ± 0.11	736 ± 50.5	444 ± 69
	**28 ± 4**	**14 ± 1**	**18 ± 2**	**15 ± 0.5**	**2.7 ± 0.1**	**1939 ± 31**	**131 ± 40**
b							
180 min	40.13 ± 2.67	26.75 ± 1.81	32.38 ± 1.3				
	**26.33 ± 4.06**	**13.33 ± 3.53**	**17.67 ± 3.84**	**10.33 ± 2.03**	**3.82 ± 0.24**	**1508 ± 107.6**	**135 ± 25.48**
210 min	35.71 ± 2.35	24.86 ± 1.86	27 ± 2.39				
	**24.67 ± 3.83**	**11.33 ± 0.33**	**15.67 ± 1.67**	**7.67 ± 1.86**	**2.81 ± 0.32**	**2144 ± 574.2**	**251.7 ± 70.34**
240 min	38.86 ± 2.18	25.57 ± 1.41	28.38 ± 2.99				
	**25.33 ± 1.67**	**13 ± 2.65**	**18 ± 2.31**	**8 ± 1**	**3.05 ± 0.06**	**1864 ± 252.6**	**262.3 ± 77.83**
270 min	40.14 ± 2.1	28.57 ± 1.56	31 ± 2.53				
	**22.33 ± 0.88**	**12.67 ± 0.88**	**16 ± 1.53**	**6.67 ± 1.33**	**3.40 ± 1.25**	**1816 ± 418.6**	**266 ± 102.3**
300 min	40.83 ± 3.72	30 ± 2.53	29 ± 4.11				
	**19.67 ± 2.85**	**12.33 ± 2.33**	**15.33 ± 2.85**	**7.33 ± 1.76**	**4.20 ± 1.77**	**1631 ± 462.3**	**246.3 ± 132.1**
330 min	39.6 ± 3.11	30.4 ± 2.98	29.86 ± 3.94				
	**21 ± 2.08**	**11.67 ± 1.76**	**15.33 ± 2.40**	**7 ± 1.73**	**4.58 ± 2.08**	**1627 ± 463.8**	**282.3 ± 150.5**
360 min	42.33 ± 3.27	31.83 ± 4.06	32.14 ± 4.49				
	**19.67 ± 3.33**	**10.67 ± 2.03**	**14 ± 2.31**	**6 ± 1.53**	**3.84 ± 1.56**	**1560 ± 312.2**	**224 ± 88.22**
390 min	41.5 ± 3.52	30.5 ± 3.23	29 ± 4.01				
	**19 ± 4.04**	**9 ± 3**	**12.33±**	**8 ± 0**	**2.42 ± 0.69**	**1811 ± 315**	**275.5 ± 94.5**
420 min	41.67 ± 2.96	30 ± 4.58	29.57 ± 3.54				
	**19.33 ± 4.18**	**9.33 ± 3.18**	**12 ± 3.51**	**8 ± 0**	**2.39 ± 0.29**	**1643 ± 32**	**261 ± 82**

Complete hemodynamic values during LPS and sham treatment and b) shows only pulmonary pressures during LPS ECMO treatment and sham treatment ECMO: systolic pulmonary pressure (SPP), diastolic pulmonary pressure (DPP), mean pulmonary pressure (MPP), pulmonary artery wedge pressure (Wedge), cardiac output (CO), systemic vascular resistance (SVR), pulmonary vascular resistance (PVR).

### 2.5 Extra‐Corporeal Membrane Oxygenation (ECMO) setup

Eleven animals, LPS ECMO animals (*n* = 8) and sham‐treated ECMO animals (*n* = 3), received ECMO for 4 h. In the LPS ECMO animals, the ECMO was connected after ARDS was confirmed with two separate blood gases taken at 15‐min intervals. ECMO equipment and use followed standard protocol (see Methods in online supplement). ECMO flow was adjusted to 75% of CO.

### 2.6 Particle flow rate (PFR) measurements and collection of EBP during *in vivo* animal studies

A customized PExA 2.0 device (PExA) was used in conjunction with mechanical ventilation and connected to the expiratory limb on the ventilator, as previously described (Broberg, Pierre, et al., 2019; Broberg et al., 2018). Particle flow rate measurements were continuously recorded throughout the experiment and presented as PFR (particles/min). Measured particles were in the diameter range of 0.41–4.55 µm. Particles were collected onto a membrane and referred to as EBP.

### 2.7 Measurements of cytokines using multiplex in plasma and bronchoalveolar lavage fluid (BALF)

Plasma samples were taken at baseline, after 30 min and every 60 min after LPS installation. BALF samples were obtained at baseline and the end with an Ambu® aScope^TM^ (Ambu). Plasma and BALF were analyzed with the cytokine multiplex kit Cytokine & Chemokine 9‐Plex Porcine ProcartaPlex™ Panel 1 (Thermo Fisher Scientific Cat. No. EPX090‐60829–901) according to the manufacturer's instructions. The kit was analyzed using a Bioplex‐200 system (BioRad). The six cytokines in the kit included: IL‐1β, IL‐6, IL‐8, IL‐10, IL‐12, and TNF‐α.

### 2.8 Measurements of proteins in EBP using Olink

The proteins in EBP were detected using Olink Multiplex inflammatory and cardiometabolic panels according to the manufacturer's website (http://www.olink.com, see supplemental Methods). The Olink Multiplex data were reported in NPX (normalized protein expression levels).

### 2.9 Histology

Baseline lung biopsies were taken from the right lower lobe through a small right thoracotomy and were also taken at the termination of the experiment through a sternotomy (i.e., after 7 h) from both the right and the left lower and upper lobes. In LPS‐treated animals (LPS ECMO animals and LPS animals) the baseline biopsies were taken before LPS administration. Biopsies were fixed, stained with hematoxylin and eosin (H&E) and scored according to standard procedures (see supplemental Methods). Brightfield images were acquired using an Olympus VS120‐S5 slide scanning system. After the end of the experiments, the gross lungs were additionally assessed for levels of hemorrhage. Using ImageJ (version 1.53a, NIH), both the area of hemorrhage and the area of the entire lung were defined and measured. The percentage of hemorrhage of the total lung was then reported.

### 2.10 Wet dry‐weight ratio

Pulmonary edema was examined by measuring the wet weight to dry weight ratio in lung tissue from the right lower lobe at baseline and from both the left and the right lower lobes at the end of the experiment.

### 2.11 Particle flow rate (PFR) measurements in four patients with ARDS and ECMO

The four patients had a mean age of 58 years (range 50–63). All patients were male. Three of the patients had COVID‐19‐induced ARDS confirmed via PCR testing of nasopharyngeal and bronchoalveolar lavage. The other patient had laboratory‐confirmed gram‐negative bacterium‐induced ARDS. The patients had symptoms of upper respiratory tract infection in 8 ± 1 days before hospital admission. The patients spent 3.6 ± 1.5 days in the hospital prior to admission to the ICU and spent 6.3 ± 3.3 days in mechanical ventilation before requiring ECMO. PFR measurements were started during the first 2 days of ECMO treatment. Thereafter, PFR measurements were done weekly until the end of the ECMO treatment. All patients had a veno‐venous ECMO connected to the superior vena cava and the femoral vein (parameters in Table 3) and chest X‐rays were performed regularly.

### 2.12 Calculations and statistics

Continuous variables were reported as mean and standard error of the mean (SEM). For histological variables and the wet‐dry ratios, values were graphically reported as mean, minimum, and maximum. Statistically significant differences between groups were tested with the Student's *t*‐test and within groups with ANOVA when data were normally distributed. The Mann–Whitney test and the Wilcoxon test were used when data were not normally distributed. All statistical analysis was performed using GraphPad Prism (version 9.0.0). Significance was defined as: *p* < 0.001 (***), *p* < 0.01 (**), *p* < 0.05 (*), and *p* > 0.05 (not significant).

## 3 RESULTS

### 3.1 Dramatically increased particle flow rate (PFR) following LPS and ECMO

**FIGURE 2:**
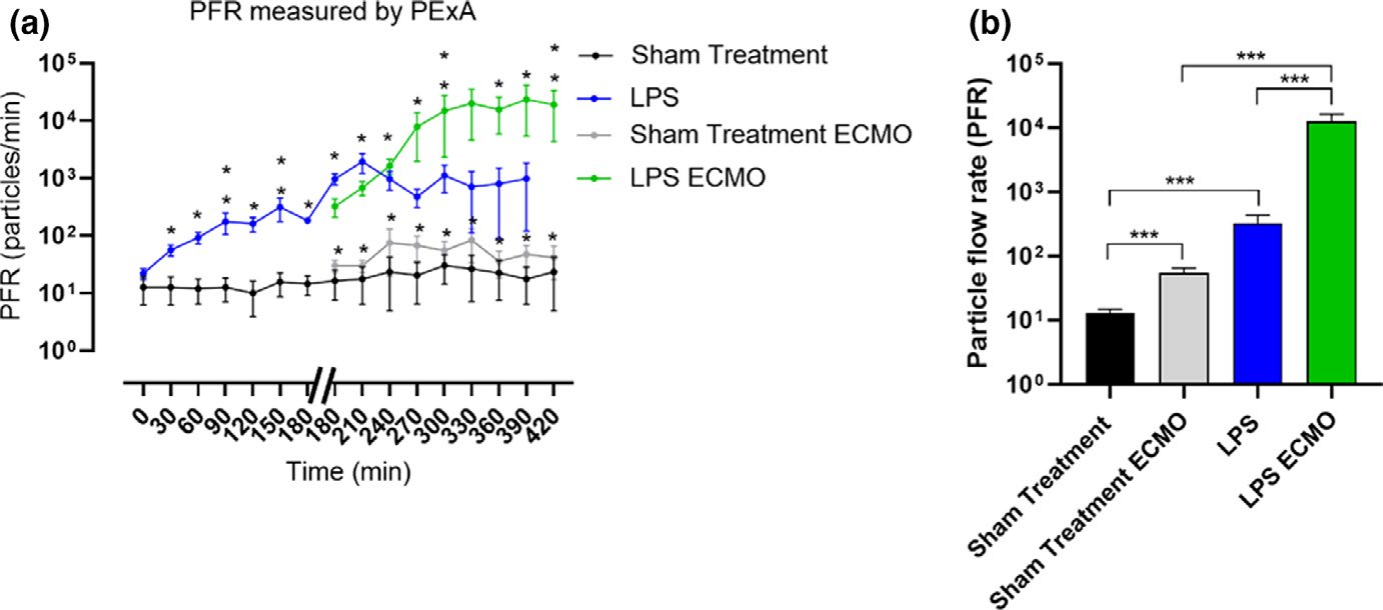
A) A timeline with particle flow rate (PFR) from the airways in the four different groups: LPS, LPS ECMO, sham treatment ECMO and sham treatment. Note how the PFR increases significantly already after 30 min after LPS treatment. Note the dramatic increase in PFR after the establishment of ECMO in LPS‐treated animals. B) The mean values of the PFR between the different treatments. Interestingly, we observed a significant increase in PFR between the sham treatment groups but also a dramatic increase in PFR between LPS treatment and LPS and ECMO treatment. Values are presented as mean ± SEM. Significance was defined as: *p* < 0.001 (***), *p* < 0.01 (**), *p* < 0.05 (*), and *p* > 0.05 (not significant)

All LPS‐treated animals developed ARDS within 180 min, as defined by two separate arterial blood gases within a 15‐min interval. Hemodynamic parameters, blood gas values, and mechanical ventilator settings and ECMO settings are shown in Tables 1, 2, 3, 4, 5. Following ARDS, eight animals were connected to VA‐ECMO and called LPS ECMO animals. In the sham‐treated ECMO animals, the VA‐ECMO was connected after 180 minutes of mechanical ventilation and saline treatment. The PFR when ECMO started in LPS‐treated animals was 324 ± 114 particles/min. The PFR continued to increase significantly over time and 30 min after the onset of ECMO, PFR was 688 ± 187 particles/min (*p* = 0.0030). Seven animals received only LPS and not ECMO (called LPS animals). These had a PFR significantly higher than sham‐treated animals at all time points. The LPS animals, however, had a lower PFR than the LPS ECMO animals. (Figure 2A). In the LPS animal cohort, only two survived more than 300 min and none of the animals survived more than 390 min.

**TABLE 3:** Blood gas values of pigs treated with (a) LPS (regular) and sham (bold) over time (b) LPS and ECMO (regular) and sham with ECMO (bold) over time

	pH	PaO₂ (mmHg)	PaCO₂ (mmHg)	Hb (g/L)	Lactate (mmol/L)	BE (mmol/L)
(a)						
Base	7.44 ± 0.02	256.4 ± 11.97	47.42 ± 2.27	105.9 ± 3.2	1.05 ± 0.09	7.76 ± 0.75
	**7.46 ± 0.04**	**240 ± 18**	**39 ± 3.0**	**103 ± 3.4**	**0.87 ± 0.03**	**3.9 ± 1.1**
30 min	7.35 ± 0.01	143 ± 9.86	55.99 ± 2.03	122 ± 5.5	1.38 ± 0.17	5.5 ± 0.55
	**7.47 ± 0.02**	**243 ± 20**	**38 ± 1.1**	**103 ± 3.1**	**0.87 ± 0.03**	**4.1 ± 0.9**
60 min	7.36 ± 0.01	150 ± 14.85	54.15 ± 1.52	135.3 ± 6.78	1.51 ± 0.19	4.79 ± 0.71
	**7.46 ± 0.02**	**256 ± 23**	**38 ± 2.7**	**103 ± 3.0**	**0.83 ± 0.09**	**3.7 ± 0.7**
90 min	7.32 ± 0.01	112.4 ± 16.82	54.38 ± 1.27	134 ± 5.68	2.13 ± 0.26	2 ± 0.95
	**7.47 ± 0.03**	**258 ± 20**	**38 ± 3.3**	**103 ± 1.2**	**0.73 ± 0.03**	**3.8 ± 0.9**
120 min	7.28 ± 0.01	78.75 ± 5.96	58.71 ± 1.98	133.6 ± 6.3	2.7 ± 0.23	0.73 ± 0.66
	**7.48 ± 0.02**	**243 ± 18**	**37 ± 2.8**	**102 ± 2.2**	**0.70 ± 0.06**	**3.7 ± 0.9**
150 min	7.26 ± 0.02	89.25 ± 17.11	61.16 ± 2.12	127 ± 4.07	2.73 ± 0.29	0.79 ± 1.1
	**7.47 ± 0.03**	**245 ± 12**	**37 ± 2.8**	**103 ± 2.1**	**0.70 ± 0.06**	**3.5 ± 0.9**
180 min	7.25 ± 0.03	99.38 ± 37.13	60.79 ± 0.79	123 ± 6	4.2 ± 0.2	−1.2 ± 2.2
	**7.46 ± 0.03**	**245 ± 15**	**38 ± 2.3**	**97 ± 7.1**	**0.63 ± 0.03**	**3.1 ± 1.1**
(b)						
180 min	7.26 ± 0.01	83.34 ± 8.55	60.95 ± 1.73	127.1 ± 3.26	3.11 ± 0.29	0.06 ± 0.77
	**7.44 ± 0.03**	**235 ± 14.84**	**42.6 ± 1.02**	**102 ± 1**	**0.8 ± 0.21**	**4.77 ± 2.37**
210 min	7.32 ± 0.01	82.41 ± 7.90	47.83 ± 1.64	113.5 ± 4.07	3.91 ± 0.66	−1.88 ± 0.98
	**7.43 ± 0.01**	**199.8 ± 54.67**	**41.55 ± 5.09**	**110.3 ± 16.42**	**1.1 ± 0.36**	**3.13 ± 3.01**
240 min	7.32 ± 0.02	89.44 ± 11.97	45.72 ± 3.25	106.8 ± 5.45	5.03 ± 0.83	−3 ± 0.80
	**7.43 ± 0.01**	**210.3 ± 47.75**	**39.98 ± 2.27**	**108.7 ± 15.5**	**1.67 ± 0.77**	**2.2 ± 2.1**
270 min	7.28 ± 0.02	80.06 ± 8.61	46.63 ± 3.41	112.2 ± 4.78	6.16 ± 0.85	−5.04 ± 0.74
	**7.42 ± 0.05**	**197.3 ± 36.79**	**41.55 ± 1.0**	**96.67 ± 10.71**	**1.53 ± 0.56**	**2.63 ± 3.45**
300 min	7.28 ± 0.02	80.72 ± 10.1	43.11 ± 2.32	116.8 ± 4.82	7.31 ± 0.95	−6.76 ± 0.78
	**7.41 ± 0.05**	**141.5 ± 22.65**	**41.73 ± 1.67**	**90 ± 9.29**	**1.4 ± 0.44**	**2 ± 3.8**
330 min	7.25 ± 0.04	72.11 ± 3.45	45.75 ± 4.36	111.4 ± 10.38	7.71 ± 1.11	−7.51 ± 1.18
	**7.40 ± 0.05**	**149.3 ± 31.89**	**42.8 ± 1.45**	**77.5 ± 14.5**	**1.43 ± 0.41**	**2.1 ± 3.6**
360 min	7.24 ± 0.03	64.5 ± 4.11	44.36 ± 3.18	111.6 ± 9.61	8.66 ± 1.35	−8.61 ± 1.11
	**7.39 ± 0.05**	**130.3 ± 27.37**	**43.7 ± 2.2**	**77 ± 8.51**	**1.57 ± 0.43**	**1.83 ± 3.43**
390 min	7.21 ± 0.04	68.25 ± 4.84	46.39 ± 3.58	107.4 ± 10.81	9.21 ± 1.50	−9.83 ± 1.2
	**7.38 ± 0.05**	**127.8 ± 28.16**	**44.9 ± 1.24**	**72.67 ± 9.49**	**1.63 ± 0.52**	**1.47 ± 3.72**
420 min	7.19 ± 0.03	65.14 ± 6.95	47.57 ± 3.82	105 ± 8.05	10.1 ± 1.72	−10.2 ± 1.07
	**7.37 ± 0.05**	**138.8 ± 25.13**	**44.73 ± 1.19**	**68.67 ± 10.87**	**1.63 ± 0.52**	**0.83 ± 3.19**

Blood gas values during LPS and sham treatment and b) shows blood gas values during LPS ECMO treatment and sham treatment ECMO: pH, partial pressure of oxygen (PaO_2_), partial pressure of carbon dioxide (PaCO_2_), hemoglobin (Hb), lactate, base excess (BE).

**TABLE 4:** Respiratory parameters of pigs treated with (a) LPS regular) and sham (bold) over time (b) LPS and ECMO (regular) and sham with ECMO (bold) over time

	MV (L/min)	PIP (cmH₂O)	PEEP (cm H₂O)	Vt (ml)	C_dyn_ (ml/cmH_2_O)	RR (breaths/min)	FiO₂ (%)	PaO₂/FiO₂ (mmHg)
(a)								
Base	8.9 ± 0.16	15.6 ± 0.57	5 ± 0	413.4 ± 11.61	39.81 ± 2.54	20.5 ± 0.53	50 ± 0	513.1 ± 23.92
	**7.4 ± 0.3**	**15 ± 0.3**	**5 ± 0**	**413 ± 13**	**40.1 ± 2.2**	**19 ± 0.7**	**50 ± 0**	**479 ± 36**
30 min	9.21 ± 0.25	16.4 ± 0.78	5 ± 0	415.6 ± 8.82	37.69 ± 2.46	21.9 ± 0.88	50 ± 0	286.1 ± 19.7
	**7.6 ± 0.4**	**15 ± 0.3**	**5 ± 0**	**397 ± 15**	**38.5 ± 2.5**	**19 ± 0.6**	**50 ± 0**	**486 ± 40**
60 min	10.03 ± 0.29	17 ± 0.46	5 ± 0	424 ± 7.16	35.72 ± 1.54	23 ± 0.89	50 ± 0	300.3 ± 29.64
	**7.6 ± 0.4**	**15 ± 0.9**	**5 ± 0**	**397 ± 15**	**39.1 ± 4.2**	**19 ± 0.6**	**50 ± 0**	**512 ± 46**
90 min	10.13 ± 0.21	18.5 ± 0.68	5 ± 0	427.3 ± 11.38	32.13 ± 1.56	23 ± 0.76	50 ± 0	224.9 ± 33.63
	**7.6 ± 0.4**	**15 ± 0.3**	**5 ± 0**	**397 ± 15**	**38.5 ± 2.5**	**19 ± 0.6**	**50 ± 0**	**517 ± 40**
120 min	10.5 ± 0.3	20.6 ± 0.84	5 ± 0	439 ± 12.15	28.7 ± 1.79	24 ± 0.85	50 ± 0	157.7 ± 11.96
	**7.6 ± 0.4**	**15 ± 0.3**	**5 ± 0**	**397 ± 15**	**38.5 ± 2.5**	**19 ± 0.6**	**50 ± 0**	**486 ± 36**
150 min	10.86 ± 0.26	21 ± 0.85	5 ± 0	447.6 ± 11.88	28.43 ± 1.63	24.6 ± 0.87	54.3 ± 4.3	172.3 ± 36.6
	**7.4 ± 0.4**	**15 ± 0.6**	**5 ± 0**	**388 ± 16**	**39.1 ± 2.7**	**19 ± 0.6**	**50 ± 0**	**489 ± 24**
180 min	11 ± 1	20 ± 0	5 ± 0	475 ± 25	31.67 ± 1.67	23 ± 3	65 ± 15	175.5 ± 97.5
	**7.4 ± 0.4**	**15 ± 0.9**	**5 ± 0**	**388 ± 16**	**38.2 ± 3.7**	**19 ± 0.6**	**50 ± 0**	**491 ± 31**
(b)								
180 min	10.69 ± 0.35	20.88 ± 0.74	5 ± 0	445.6 ± 11.13	28.43 ± 1.44	24.13 ± 0.88	57.5 ± 4.9	
	**8.17 ± 0.44**	**16.8 ± 0.80**	**5 ± 0**	**425.3 ± 16.38**	**36.2 ± 1.2**	**18.67 ± 0.33**	**50 ± 0**	
210 min	10.88 ± 0.35	21.25 ± 0.45	5 ± 0	446.4 ± 11.47	27.62 ± 1.08	24.13 ± 0.88	57.5 ± 4.9	
	**8.17 ± 0.44**	**17.33 ± 1.33**	**5 ± 0**	**425.3 ± 16.38**	**35.0 ± 2.4**	**18.67 ± 0.33**	**50 ± 0**	
240 min	10.94 ± 0.37	22.13 ± 0.64	5 ± 0	459.6 ± 20.87	26.92 ± 0.98	24.13 ± 0.93	57.5 ± 4.9	
	**8.17 ± 0.44**	**17.33 ± 1.33**	**5 ± 0**	**425.3 ± 16.38**	**35.0 ± 2.4**	**18.67 ± 0.33**	**50 ± 0**	
270 min	10.94 ± 0.37	22.38 ± 0.60	5 ± 0	453 ± 21.98	26.09 ± 0.87	24.13 ± 0.93	58.1 ± 5.3	
	**8.17 ± 0.44**	**17.33 ± 1.33**	**5 ± 0**	**425.3 ± 16.38**	**35.0 ± 2.4**	**18.67 ± 0.33**	**50 ± 0**	
300 min	10.88 ± 0.36	23 ± 0.91	5 ± 0	464 ± 20.89	26.05 ± 1.26	24.13 ± 0.93	58.1 ± 5.3	
	**8.17 ± 0.44**	**17 ± 1.53**	**5 ± 0**	**425.3 ± 16.38**	**36.3 ± 3.3**	**18.67 ± 0.33**	**50 ± 0**	
330 min	10.71 ± 0.38	22.86 ± 1.32	5 ± 0	438.1 ± 9.52	25.16 ± 1.53	24.71 ± 0.84	55 ± 5	
	**8.17 ± 0.44**	**18.33 ± 0.88**	**5 ± 0**	**412 ± 28**	**31.0 ± 2.1**	**19.33 ± 0.33**	**50 ± 0**	
360 min	10.71 ± 0.38	23 ± 1.11	5 ± 0	442.6 ± 10.25	25.06 ± 1.47	25.14 ± 1.1	55 ± 5	
	**8.17 ± 0.44**	**18 ± 1.16**	**5 ± 0**	**412 ± 28**	**32.1 ± 3.1**	**19.33 ± 0.33**	**50 ± 0**	
390 min	10.71 ± 0.38	24.86 ± 1.93	5 ± 0	434 ± 11.68	22.86 ± 1.86	25.14 ± 1.1	55 ± 5	
	**8.33 ± 0.33**	**18.33 ± 1.20**	**5 ± 0**	**417 ± 23.43**	**31.8 ± 3.4**	**19.67 ± 0.67**	**50 ± 0**	
420 min	10.71 ± 0.38	25.14 ± 2.06	5 ± 0	432 ± 10.63	22.61 ± 1.98	25.14 ± 1.1	59.3 ± 6	
	**8.33 ± 0.33**	**18.33 ± 1.20**	**5 ± 0**	**417 ± 23.43**	**31.8 ± 3.4**	**19.67 ± 0.67**	**50 ± 0**	

Respiratory parameters with volume‐controlled ventilation during LPS and sham treatment and b) shows respiratory parameters with volume‐controlled ventilation during LPS ECMO treatment and sham treatment ECMO: minute volume (MV), peak inspiratory pressure (PIP), positive end‐expiratory pressure (PEEP), tidal volume (Vt), dynamic compliance (Cdyn), respiratory rate (RR), fraction of inspired oxygen (FiO_2_), partial pressure of oxygen/fraction of inspired oxygen (PaO_2_/FiO_2_).

**TABLE 5:** ECMO values of pigs treated with LPS and ECMO (regular) and sham with ECMO (bold) over time

	Flow	Rpm	Pa (mmHg)	Pv (mmHg)	O2	Sweep
210 min	1.88 ± 0.18	1989 ± 80.34	104.6 ± 10.87	18.29 ± 4.10	51.25 ± 1.25	1.74 ± 0.04
	**1.79 ± 0.52**	**1730 ± 335**	**149 ± 34.12**		**53.33 ± 3.33**	**1 ± 0.29**
240 min	1.95 ± 0.17	2033 ± 73.38	106.3 ± 11.37	19.57 ± 4.12	51.25 ± 1.25	1.74 ± 0.04
	**1.773 ± 0.52**	**1770 ± 355.1**	**157 ± 38.53**		**60 ± 10**	**1.17 ± 0.67**
270 min	2.91 ± 0.42	2475 ± 153.9	130.9 ± 12.95	3.71 ± 6.27	53.75 ± 2.63	1.86 ± 0.13
	**3.08 ± 0.08**	**2260 ± 110**	**188.7 ± 14.44**		**60 ± 10**	**1.17 ± 0.67**
300 min	3.30 ± 0.28	2726 ± 56.25	138.5 ± 9.32	−7.43 ± 7.06	53.75 ± 2.63	1.96 ± 0.22
	**3.03 ± 0.26**	**2260 ± 110**	**175 ± 17.79**		**56.67 ± 6.67**	**1.17 ± 0.67**
330 min	3.60 ± 0.22	2777 ± 50.32	137.9 ± 5.41	−8 ± 6.72	57.14 ± 7.14	1.96 ± 0.26
	**3.04 ± 0.27**	**2260 ± 110**	**174.7 ± 16.42**		**56.67 ± 6.67**	**1.17 ± 0.67**
360 min	3.43 ± 0.2	2784 ± 60.98	128.9 ± 6.71	−24.43 ± 14	60 ± 7.24	2.14 ± 0.29
	**3.27 ± 0.18**	**2260 ± 110**	**180 ± 7.51**		**56.67 ± 6.67**	**1.17 ± 0.67**
390 min	3.67 ± 0.24	2820 ± 67.89	137.1 ± 8.24	−15.71 ± 12.15	60 ± 7.24	2.19 ± 0.28
	**3.29 ± 0.17**	**2260 ± 110**	**178.7 ± 8.35**		**56.67 ± 6.67**	**1.17 ± 0.67**
420 min	3.23 ± 0.22	2789 ± 60.49	126.9 ± 9.04	−28.71 ± 15.25	64.29 ± 7.51	2.19 ± 0.28
	**3.33 ± 0.20**	**2260 ± 110**	**172.3 ± 6.74**		**56.67 ± 6.67**	**1.17 ± 0.67**

ECMO values during LPS ECMO treatment and sham treatment ECMO: Flow, rounds per minute (Rpm), arterial pressure in cannula (Pa), venous pressure in cannula (Pv), oxygen delivery (O_2_), sweep flow (Sweep).

PFR was compared between the four animal groups and observed a significant increase in PFR not only between the sham‐treated animals with and without ECMO, but also between LPS animals and LPS ECMO animals (Figure 2B).

### 3.2 Macroscopic assessment of hemorrhage in the lung parenchyma showed more widespread areas in the LPS ECMO animals

**FIGURE 3:**
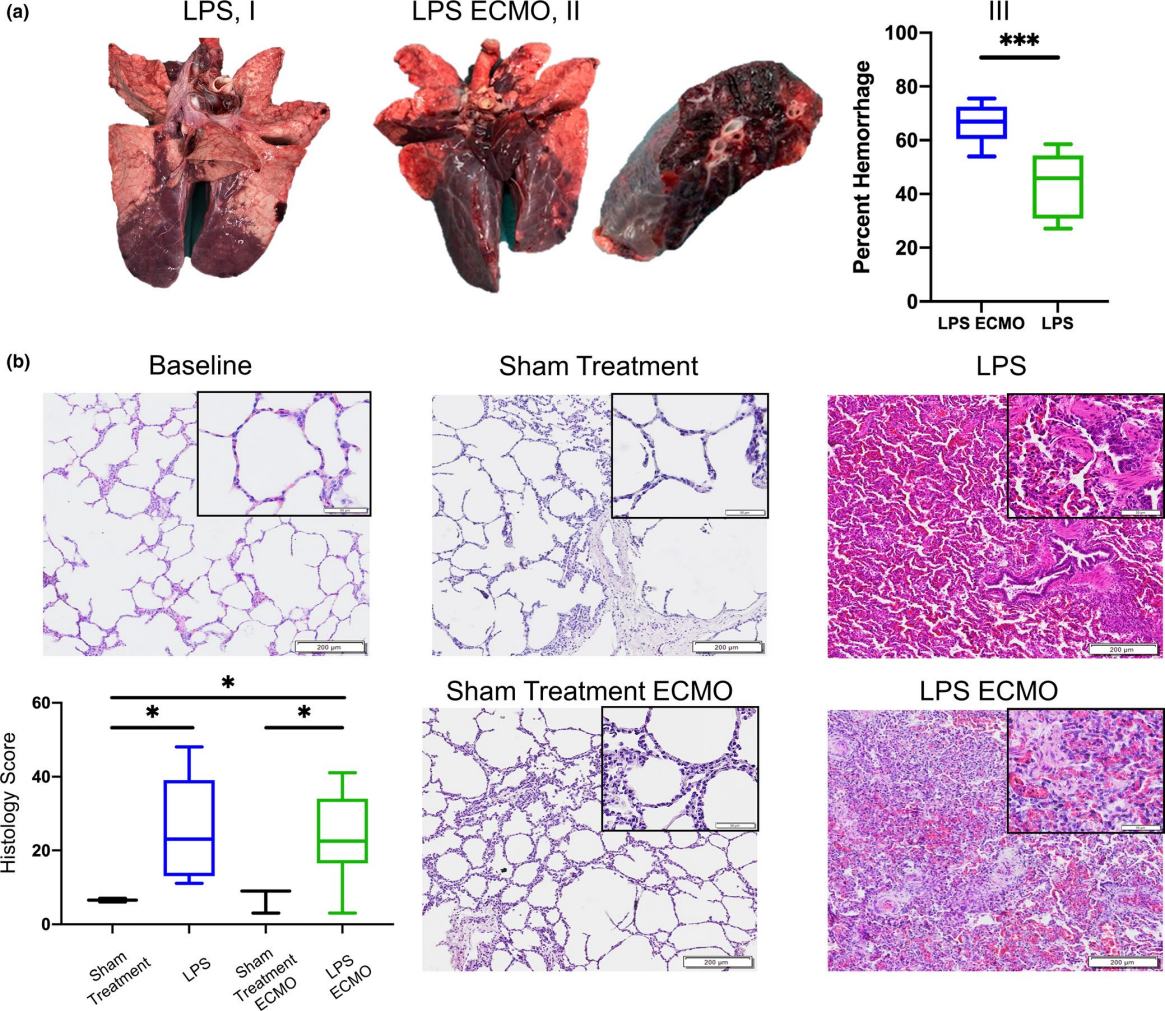
A) Macroscopic changes in lung tissue obtained 7 h after LPS administration (LPS, I) and also after 4 h of ECMO following LPS (LPS ECMO, II). The LPS ECMO lungs show greater evidence of widespread areas of severe hemorrhage, as quantified macroscopically in III. B) Lung sections (I, II, III, IV, V) stained by H&E, obtained from different treatment conditions in the respective magnifications (40× 100× and 200×). I) Baseline tissue obtained at the start of the experiment. II) sham‐treated animals: tissue obtained after 7 h of sham treatment (with saline and mechanical ventilation) didn't show any structural damage. III) LPS animals: Tissue obtained approximately 7 h after LPS administration with mechanical ventilation showed evidence of hemorrhage, presence of inflammatory cells and structural damage. IV) Sham‐treated ECMO animals: tissue obtained 7 hours after sham treatment with saline and mechanical ventilation including 4 h of ECMO showing a mild thickening of the alveolar wall. V) LPS ECMO animals: Tissue obtained 7 h after LPS including 4 h of ECMO showed evidence of severe hemorrhage, presence of inflammatory cells, and structural damage

At the experiment's end, the parenchyma of the harvested lung was macroscopically assessed. Areas of hemorrhage and thrombosis were assessed visually. In sham treatment animals, no areas of hemorrhage or thrombosis were found, whereas large areas of hemorrhage and thrombosis were found in the lung parenchyma of both LPS and LPS ECMO animals. The LPS ECMO animals, however, had more macroscopic hemorrhage, with an average of 66.6% ± 2.8 hemorrhage (max 75.6%, min 54.0%) (Figure 3A). This was significantly greater than the LPS animals which had 43.3% ± 3.4 hemorrhage (max 58.6%, min 27.2%; *p* = 0.0002).

### 3.3 Histological characteristics of ARDS after LPS and ECMO

H&E stained lung biopsies from the lower lobes confirmed the onset of severe lung damage. Lung tissue taken before LPS administration appeared healthy. However, 7 h later, in both the LPS and LPS ECMO cohorts, significant infiltration of immune cells and signs of diffuse alveolar damage could be observed, including thickening of the alveolar capillary barrier with intra‐alveolar hemorrhage and edema. Significant increases in lung damage were observed in LPS animals and LPS ECMO animals compared to sham‐treated animals, as assessed through histological scoring. No significant differences in histological scoring were observed between sham‐treated animals and sham‐treated ECMO animals, nor between LPS animals and LPS ECMO animals.

**FIGURE 4:**
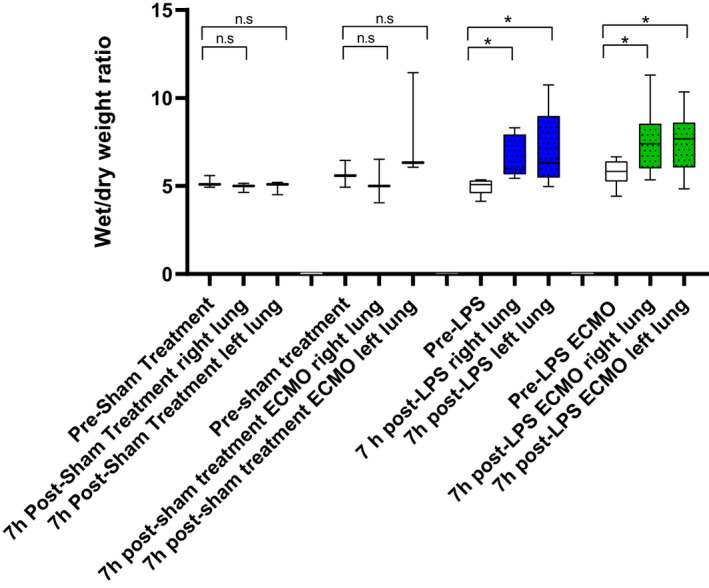
The figure shows wet/dry weight ratio from lung tissue at baseline and at 7 h post‐LPS ECMO and post‐LPS treatment compared to sham‐treated animals and sham‐treated ECMO animals. Wet/dry weight ratio shows a significant increase in edema after LPS administration in LPS and LPS ECMO animals compared to baseline biopsies and sham‐treated animals. ECMO animals (both sham and LPS treated) showed a tendency toward more edema compared to animals not treated in ECMO, however the differences were not significant

The lung tissue wet/dry weight ratios were measured at baseline and at 7 h post‐LPS administration in LPS animals and in LPS ECMO animals. These were compared to sham‐treated animals with and without ECMO. There were significant increases in edema in LPS animals and LPS ECMO animals compared to baseline biopsies and sham‐treated animals with and without ECMO (Figure 4). A tendency toward increased edema was seen in sham‐treated ECMO animals compared to sham‐treated animals, and in LPS ECMO animals compared to LPS animals, however, these tendencies were not significant (Figure 4).

### 3.4 Cytokine response over time following LPS administration and ECMO

**FIGURE 5:**
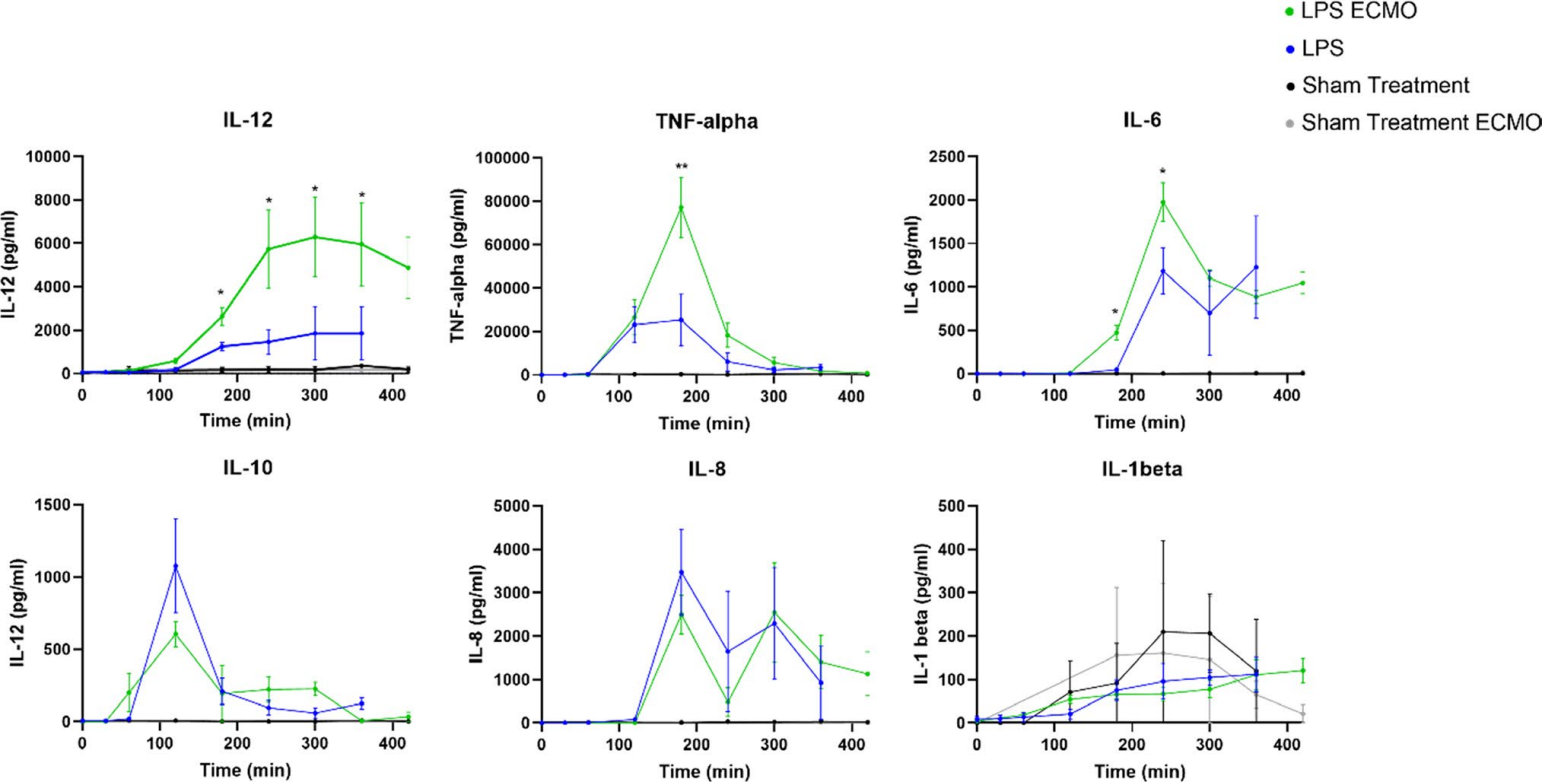
The concentration of IL‐1beta (interleukin‐1 beta), IL‐10 (interleukin‐10), IL‐12 (interleukin‐12), IL‐8 (interleukin‐8), IL‐6 (interleukin‐6), and TNF‐alpha (tumor necrosis factor‐alpha), in plasma measured by multiplex at baseline and at different time points after LPS administration and compared between LPS and LPS ECMO group. Significance was defined as *p* < 0.001 (***), *p* < 0.01 (**), *p* < 0.05 (*), and *p* > 0.05 (not significant)

In order to monitor the inflammatory response after LPS administration and ECMO treatment and see if changes in plasma markers changes coincide with increased PFR, cytokine concentrations were measured in plasma collected every hour. Significant increases in pro‐inflammatory cytokines IL‐6, IL‐12, and TNF‐α were seen in the LPS ECMO animals compared to LPS animals. IL‐10 was elevated early in both LPS animals and LPS ECMO animals (around 100 min), but reached higher peak levels in the LPS animals. The sham‐treated animals had significantly lower cytokine levels at all time points compared to both LPS and LPS ECMO animals with the exception of IL‐1β (Figure 5).

### 3.5 Pro‐inflammatory cytokines increase in BALF following LPS administration and ECMO

**FIGURE 6:**
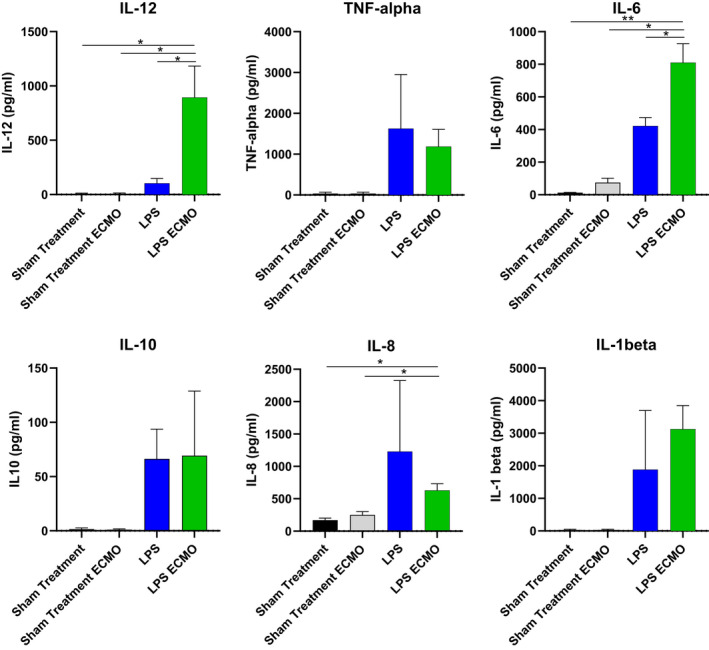
The concentration of cytokines IL‐1beta (interleukin‐1 beta), IL‐10 (interleukin‐10), IL‐12 (interleukin‐12), IL‐8 (interleukin‐8), IL‐6 (interleukin‐6), and TNF‐alpha (tumor necrosis factor‐alpha), in bronchoalveolar lavage fluid (BALF) measured by multiplex at baseline and at the end of the experiment. Comparison is made between LPS and LPS ECMO group. Significance was defined as *p* < 0.001 (***), *p* < 0.01 (**), *p* < 0.05 (*), and *p* > 0.05 (not significant)

Bronchoscopy with BALF was performed before LPS administration at baseline and the end of the experiment in all four groups. The concentration of all cytokines analyzed increased significantly in both LPS animals and LPS ECMO animals compared to baseline and sham‐treated animals. The concentrations of IL‐6 and IL‐12 increased significantly in the LPS ECMO animals compared to the LPS animals. Between sham treatments, there was a significant rise in IL‐6 in the sham‐treated ECMO animals relative to those without ECMO (Figure 6).

### 3.6 Measurements of proteins in EBP using Olink proteomics

**FIGURE 7:**
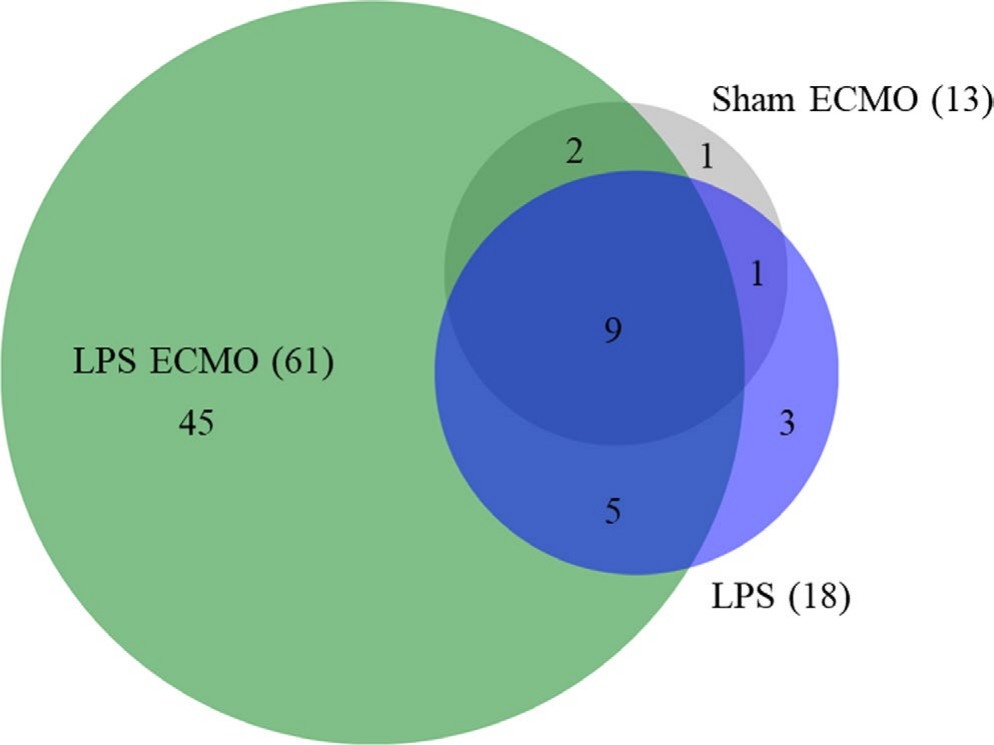
Proteins detected in EBP using Olink proteomics. From the inflammatory and cardiometabolic panels offered by Olink, proteins were detected in each of the animal groups. Values in parentheses indicate the total number of proteins found in that group regardless of overlap with those proteins found in another group. Those found in the LPS ECMO and LPS animals included known ALI and ARDS biomarkers, such as FAS ligand, vascular endothelial growth factor‐A (VEGF‐A), MCP‐1, C‐X‐C motif chemokine 10 (CXCL10), and MMP‐1

EBPs were analyzed from baseline, following ARDS development and at the end of the time course using Olink proteomics due to the method's sensitivity to small protein amounts. The number of targets detected in each cohort is shown in Figure 7. Many of the detected proteins in the LPS animals and LPS ECMO animals were all well‐recognized biomarkers for ALI and ARDS, including FAS ligand, vascular endothelial growth factor‐A (VEGF‐A), MCP‐1, C‐X‐C motif chemokine 10 (CXCL10), and MMP‐1 (Bless et al., 2000; Hergrueter et al., 2011; Lang et al., 2017).

### 3.7 Pulmonary recovery resulted in a decrease in PFR in contrast to an increase in ECMO patients who deteriorated

**FIGURE 8:**
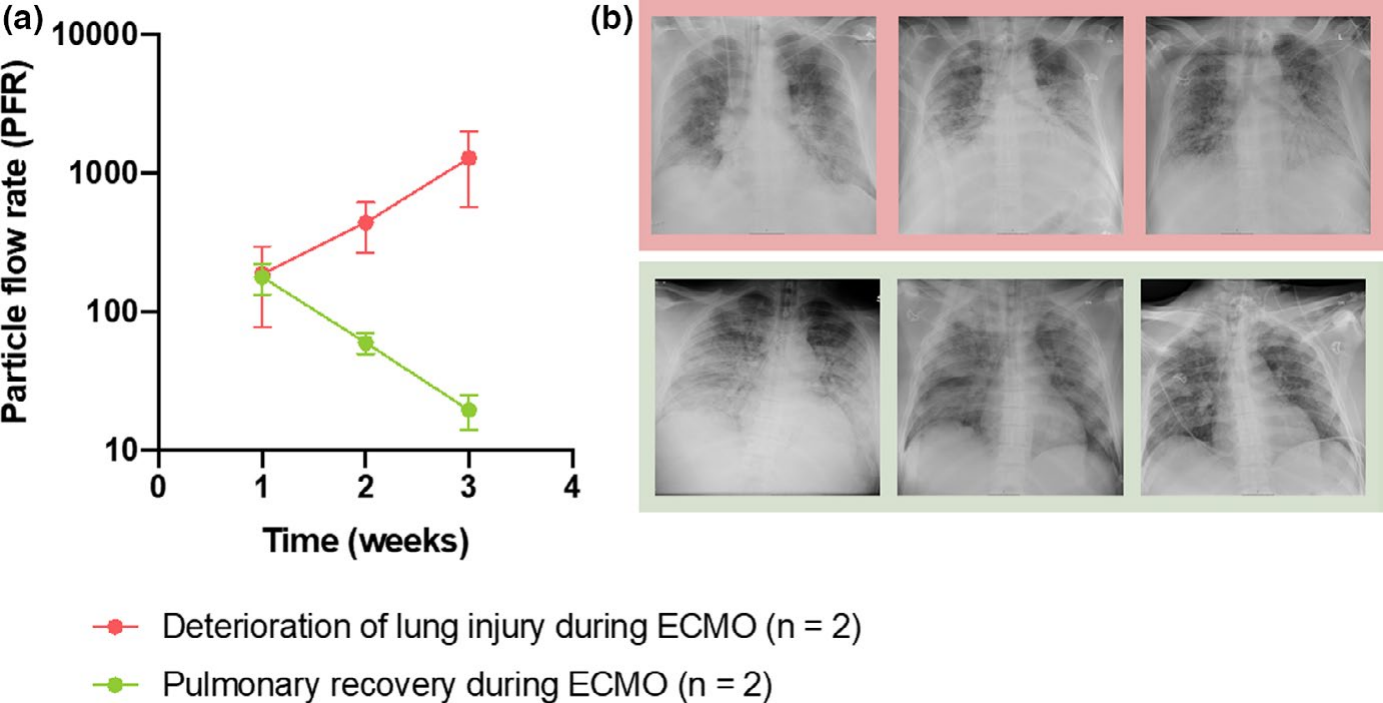
A) PFR was measured in four patients with ECMO. Three of the four patients had COVID‐19‐induced ARDS. Two of the patients recovered and could be taken off ECMO and showed a decrease in PFR, whereas two patients deteriorated in lung function during the ECMO treatment and showed an increase in PFR over time. One of the patients died and one was still on ECMO after 8 weeks without any signs of pulmonary recovery. B) The X‐rays of the patient who deteriorated in condition (top) and those of the patient who improved (bottom) mirror the changes in PFR over time. From left to right, X‐rays were taken at the time of intubation, at the start of ECMO, and at the time patients were weaned from ECMO

Having confirmed that PFR monitoring could be used in a porcine model of ARDS and ECMO, we enrolled a small cohort of patients to test the clinical application of this approach (Table 7). Three of the four patients had COVID‐19‐induced ARDS, whereas one patient had gram‐positive bacterium‐induced ARDS. All four patients had a similar PFR at the time of ECMO treatment initiation (Figure 8A). However, two recovered and could be weaned from ECMO, over which time they showed a decrease in PFR together with an increase in tidal volume and lung compliance (Table 9) along with improved gas‐exchange (Table 8) and improvement in their chest x‐rays (Figure 8B). Two patients deteriorated in lung function during the ECMO treatment and showed an increase in PFR over this time (Figure 8A). These patients similarly had a deterioration in their gas‐exchange and overall pulmonary function (Tables 8 and 9) and worsening chest X‐rays (Figure 8B). One of the patients died after 3 weeks on ECMO and one patient was still on ECMO after 8 weeks without any signs of recovery of lung function according to the CT scans. The patients had a mean D‐dimer concentration in plasma of 28.5 ± 11.5 mg/L at the initiation of ECMO and 7.8 ± 0.9 mg/L at the time of the first PExA measurement, and an IL‐6 concentration in plasma of 3658 ± 2572 ng/L at the initiation of ECMO and 428 ± 265 ng/L at the time of the first PExA measurement.

**TABLE 6:** Physiologic status of patients that deteriorated over time (regular) and improved over time (bold)

	Sat (%)	HR (bpm)	SBP (mmHg)	DBP (mmHg)	MAP (mmHg)	CVP (mmHg)	Temp (°C)
Start	95.5 ± 2.5	83.5 ± 11.5	137 ± 10	66 ± 11	85.5 ± 10.5	7 ± 3	37.35 ± 0.55
	**92.5 ± 6.5**	**85.5 ± 1.5**	**126 ± 4**	**68 ± 11**	**88.5 ± 8.5**	**14 ± 3**	**36.95 ± 0.05**
2 weeks ECMO	94.5 ± 1.5	86 ± 10	126.5 ± 8.5	60.5 ± 8.5	79.5 ± 3.5	7.5 ± 1.5	37.4 ± 0.5
	**97.5 ± 2.5**	**88 ± 6**	**108 ± 8**	**59 ± 4**	**76 ± 5**	**11.5 ± 1.5**	**36.5 ± 0.1**
3 Weeks ECMO	94.5 ± 2.5	86 ± 13	117.5 ± 10.5	56.5 ± 4.5	74.5 ± 7.5	6 ± 3	37.55 ± 0.45
	**94 ± 6**	**86 ± 11**	**124 ± 13**	**66.5 ± 6.5**	**86.5 ± 8.5**	**10 ± 4**	**37.1 ± 0.1**

The physiologic status of the human patients who both improved over time and those that deteriorated over time following the initiation of ECMO (start), 2 weeks following ECMO initiation (2 weeks ECMO) and then 3 weeks after (3 weeks ECMO). oxygen saturation (Sat), heart rate (HR), systolic blood pressure (SBP), diastolic blood pressure (DBP), mean arterial pressure (MAP), central venous pressure (CVP), temperature (Temp).

**TABLE 7:** Blood gas values of patients that deteriorated over time (regular) and improved over time (bold)

	pH	PaO_2_ (mmHg)	PaCO_2_ (mmHg)	Hb (g/L)	Lactate (mmol/L)	BE (mmol/L)
Start	7.475 ± 0.055	8.15 ± 1.45	5.5 ± 0.1	99.5 ± 20.5	1.85 ± 0.15	6.2 ± 3.7
	**7.355 ± 0.065**	**8.4 ± 0.1**	**7.15 ± 0.75**	**129.5 ± 22.5**	**1.95 ± 0.15**	**3.75 ± 2.45**
2 weeks ECMO	7.415 ± 0.025	7.55 ± 0.05	6.45 ± 0.85	103.5 ± 6.5	1.95 ± 0.65	6.4 ± 6
	**7.445 ± 0.015**	**11.55 ± 1.45**	**6.5 ± 0.5**	**117.5 ± 13.5**	**1.5 ± 0.4**	**8.7 ± 3.8**
3 Weeks ECMO	7.34 ± 0.01	9.4 ± 1.4	6.95 ± 1.15	107 ± 8	3.25 ± 2.45	2.05 ± 3.75
	**7.37 ± 0.11**	**13.55 ± 1.25**	**5 ± 0**	**94.5 ± 4.5**	**1.55 ± 0.35**	**1.55 ± 2.55**

The blood gas values for the human patients. pH, partial pressure of oxygen (PaO_2_), partial pressure of carbon dioxide (PaCO_2_), hemoglobin (Hb), lactate, base excess (BE).

**TABLE 8:** Respiratory parameters of patients that deteriorated over time (regular) and improved over time (bold)

	MV (L/min)	PIP (cmH_2_O)	PEEP (cmH_2_O)	Vt (ml)	C_dyn_ (ml/cmH_2_O)	RR (breaths/min)	FiO_2_ (%)
Start	7.05 ± 0.25	25 ± 3	10 ± 0	473.5 ± 73.5	31 ± 1	23.5 ± 3.5	30 ± 0
	**7.35 ± 3.25**	**18.5 ± 1.5**	**11.5 ± 1.5**	**366 ± 208**	**35.5 ± 5.5**	**20 ± 0**	**70 ± 30**
2 weeks ECMO	10.55 ± 0.85	26.5 ± 3.5	9 ± 1	420 ± 45	26.5 ± 2.5	14.5 ± 5.5	42.5 ± 2.5
	**7.55 ± 0.65**	**24.5 ± 0.5**	**11 ± 1**	**499 ± 240**	**37.5 ± 18.5**	**16.5 ± 5.5**	**35 ± 5**
3 Weeks ECMO	9.4 ± 1	27 ± 3	9 ± 1	318 ± 37	18 ± 4	20.5 ± 0.5	45 ± 5
	**9.85 ± 5.05**	**21.5 ± 1.5**	**10 ± 0**	**586.5 ± 70.5**	**35.5 ± 16.5**	**21 ± 1**	**30 ± 0**

The respiratory parameters of the patients. minute volume (MV), peak inspiratory pressure (PIP), positive end‐expiratory pressure (PEEP), tidal volume (Vt), dynamic compliance (Cdyn), respiratory rate (RR), fraction of inspired oxygen (FiO_2_).

**TABLE 9:** ECMO parameters of patients that deteriorated over time (regular) and improved over time (bold)

	Flow (L/min)	RPM	Pa (mmHg)	Pv (mmHg)	O_2_ (%)	Sweep (L/min)
Start	4 ± 0	2902.5 ± 2.5	138.5 ± 1.5	−57.5 ± 0.5	100 ± 0	4.5 ± 0.5
	**4 ± 0.1**	**3095 ± 45**	**152 ± 19**	**−73 ± 0**	**95 ± 5**	**3.75 ± 0.25**
2 weeks ECMO	4.25 ± 0.15	2920 ± 20	151 ± 4	−57.5 ± 1.5	100 ± 0	7.5 ± 0.5
	**4 ± 0.1**	**3060 ± 60**	**156 ± 14**	**−67 ± 8**	**100 ± 0**	**5.75 ± 0.75**
3 Weeks ECMO	4.1 ± 0	2875 ± 25	143 ± 2	−38 ± 1	100 ± 0	8.25 ± 0.25
	**3.6 ± 0.9**	**2650 ± 350**	**126.5 ± 19.5**	**−44 ± 25**	**50 ± 0**	**2.25 ± 1.25**

The ECMO parameters employed during treatment with ECMO on the human patients. Flow, rounds per minute (Rpm), arterial pressure in cannula (Pa), venous pressure in cannula (Pv), oxygen delivery (O_2_), sweep flow (Sweep).

## 4 DISCUSSION

ECMO support can facilitate lung‐protective ventilation in patients with severe ARDS, such as that induced by COVID‐19 (Falk et al., 2019; Finney, 2014). When used with protective mechanical ventilation, it can improve outcomes for patients (Finney, 2014; Morris et al., 1994; Zapol et al., 1979). Since the outbreak of COVID‐19, as many as 1752 COVID‐19 patients have been treated with ECMO with a discharge from ECMO/survival rate of 55% according to the ELSO register (July 2020) (https://www.elso.org/COVID19.aspx). However, limitations exist given that previous studies have shown that ECMO may aggravate an existing lung injury and has the potential to lead to complications such as pulmonary hemorrhage, thromboembolic disease, and hemorrhagic infarction (Lee et al., 2018; Millar et al., 2016; Robba et al., 2017). Its use in ARDS with septic shock has at times not been recommended as a standard course of therapy (Myers et al., 2020; Rhodes et al., 2017).

As a life‐prolonging measure, ECMO does not directly ameliorate underlying lung injury. Its use transfers the responsibility of oxygenation from native lung tissue to an extracorporeal membrane, providing the injured lung an opportunity to recover. Thus a measure of the condition of the native lung is needed until weaning from ECMO is possible according to current standards (Broberg, Pierre, et al., 2019; Broberg et al., 2018; Shekar et al., 2020). A bedside measure of pulmonary state would be of great clinical value given the significant risks of imaging techniques which require transport of the patient. Currently under accepted care, the decision to transport a patient to CT imaging is taken based on clinical progression or deterioration of the patient on a non‐regulated basis. As a result, in this study, PFR measurements were taken continuously during the porcine model as anesthesiologists monitored the animals continuously and were taken on a weekly basis in human patients to determine the clinical relevancy of using PFR as a measure of changing in pulmonary status.

In this study, to examine the state of lungs in all individuals on ECMO, we show that PFR increases following the initiation of ECMO in an LPS ECMO porcine model to a greater extent than ECMO in a sham‐treated animal and furthermore demonstrate human patients who improved on ECMO had lower PFRs and those who deteriorated had higher PFRs.

The motivation for this work was to investigate if PFR could serve as a tool to measure the progress of lung injury and recovery over time during ECMO treatment. Previously, we have shown that PFR could be used as an early indicator for ARDS (Stenlo et al., 2020). We also showed that lung transplanted patients with primary graft dysfunction (PGD), a form of acute lung injury resembling ARDS, exhibit increased PFR compared to patients without PGD (Broberg, Hyllen et al., 2019). To explore the relationship of ECMO and PFR specifically, this study placed animals on ECMO after confirmed ARDS. Histologically confirmed acute lung injury coincided with dramatically increased PFR over all time points in LPS‐treated animals. PFR was significantly increased following ECMO establishment. When comparing LPS ECMO animals to those with LPS alone, PFR was increased along with the amount of hemorrhage macroscopically observed in the lungs. The concurrence of high PFR with greater lung injury demonstrates that the particles can be reflective of the worsening condition of the lungs. This rise in PFR was also seen following the start ECMO in sham ECMO animals relative to their non‐ECMO correlates, but PFR was significantly greater in LPS ECMO animals compared to sham‐treated ECMO animals. From this, it can be inferred that the induced ARDS state correlates with a higher PFR that can be monitored during ECMO treatment.

During the experiment, we sampled plasma and BALF. In the plasma, we found significant increases in TNF‐α, IL‐6, and IL‐12 between 60 and 180 min in all animals who received LPS, which is similar to the cytokine profiles in other porcine models of ARDS shown by us and others (Stenlo et al., 2020; Wyns et al., 2015). The rapid and massive increases of TNF‐α, IL‐6, and IL‐10 that were observed in our model have been shown to be linked with mortality in ARDS patients and COVID‐19 patients (Han et al., 2020; Huang et al., 2020; Liu et al., 2017). The PFR increased 30–90 min before an increase in cytokines could be detected in plasma, indicating that real‐time monitoring of PFR can be used as a clinical indicator for earlier detection of ARDS, in line with previous findings (Stenlo et al., 2020). Elevated IL‐6 concentrations during ECMO treatment have been associated with parenchymal damage in animal models (Shi et al., 2014), and also connected to worse outcomes in ECMO patients (Risnes et al., 2008). Here we show a significant increase in IL‐6 concentrations in plasma and BALF in LPS ECMO animals compared to sham‐treated ECMO animals. This coincides with the increased PFR seen in LPS ECMO animals relative to sham‐treated ECMO animals as well. Furthermore, IL‐6 was significantly increased in LPS ECMO animals compared to LPS animals as well as IL‐12 and TNF‐α, denoting a correlation of ECMO itself with an inflammatory response in the lung. The histology also showed more macroscopic widespread areas of hemorrhage and thrombosis in these animals. The increase in IL‐6 and lung injury occurred not only in the LPS animals but more so in the LPS ECMO animal, and correlated with a significant increase in PFR.

The increase in these inflammatory markers is important given the association between the cytokines and ARDS as noted in the literature. TNF‐α has been discussed as a proinflammatory cytokine downstream of pattern recognition receptors implicated in the pathogenesis of ARDS (Takeuchi and Akira, 2010; Butt et al., 2016). When studied in the context of ARDS, it has been cited as having higher levels in those patients who do not survive, which has led to it being posited as a potential biomarker (Butt et al., 2016). TNF‐α specifically reflects lung injury severity rather than diagnosis as it is higher in patients with ARDS even compared to those with severe pneumonia, as reported by Bauer (2000).

IL‐6 is also noted as a promising biomarker when predicting both morbidity and mortality in ARDS. In clinical studies, it has been increased in both plasma and BAL samples in patients who do not survive (Butt et al, 2016). Furthermore, it has been correlated with a poorer oxygenation index and with longer times spent on a ventilator (Meduri et al, 1995; Agrawal et al, 2012). In patients who acquired ARDS following severe TBI, there was an increase in IL‐6 relative to those patients without ARDS, which led to the conclusion of the authors that the cytokine was associated with ARDS (Aisiku et al, 2016).

Alveolar M1 macrophages secrete pro‐inflammatory cytokines including IL‐12 (Yang et al., 2018). These macrophages play an important role in mediating the inflammatory response in ARDS and will also produce TNF‐α to activate neutrophils and recruit more inflammatory cells to the alveoli.

Given the role these cytokines play in lung injury and the relationship we have demonstrated here between the pattern of PFR increasing as the cytokines increase in states of LPS‐induced lung injury, we believe that PFR is a measure of the severity of damage to the lungs. PFR is higher in LPS animals and higher still in LPS ECMO animals.

Furthermore, proteins associated with acute lung injury and ARDS were detected in the EBP. These proteins included FAS ligand, VEGF‐A, MCP‐1, CXCL10, and MMP‐1 and were found to be significantly higher within both the LPS animals and LPS ECMO animals. The finding that increased PFR coincided with damage to the lungs supports the conclusion that PFR can be used as a measurement of increasing lung injury over time.

Studying lung recovery is prohibitive in this animal model due to the intensive care that would be needed to allow for lung recovery over days and weeks. Therefore, PFR could not be tracked in our animal model during lung recovery and potential repair. To explore the utility of PFR in human patients, we studied four patients with ECMO treatment over a time period of 3 weeks. All four patients had a similar PFR level at the start of the ECMO treatment. In the two patients who recovered in lung function during ECMO treatment, there was a decrease in PFR over time. This occurred concurrently with an increased tidal volume, increased lung compliance, and improved air content on chest X‐ray. In the two whose lung function deteriorated during ECMO treatment, there was a significant increase in PFR over time. At these points, the patients were found to have worsening in gas‐exchange and overall pulmonary function. Additionally, their chest X‐rays were considered to be reflective of a worsening condition. As previously reported (Broberg, Pierre, et al., 2019; Broberg et al., 2018), higher tidal volumes yield a larger number of particles. Resultantly, as those patients whose condition deteriorated had lower tidal volumes, one might expect that the PFR would accordingly decrease if the tidal volume was the main factor determining PFR. Instead, we observed that these declining patients had higher PFRs as their lung condition worsened. These results demonstrate that PFR mirrors the clinical state of lung function. Trends in PFR could thus be used by clinicians to predict the progression of patients put on ECMO. In the LPS porcine model, we measured PFR in the early phase of ARDS as the animals have an initial cytokine storm. Both human patients and the LPS porcine model showed the same patterns of PFR following ARDS, although the absolute values of the particles are higher in the animal model likely because the experimental set up allowed for the initial cytokine storm to be captured.

While the results of this study are encouraging for the use of PFR measured by a bedside device in monitoring both recovery and deterioration of lung function, this study included a relatively small number of animals and patients. Nonetheless, our results indicate the potential of the technique as a complementary method to those currently used in the clinic to generate further important knowledge on the pathophysiology of the lung in the intensive care setting.

## 5 CONCLUSIONS

Recovery and deterioration of lung function were reflected in PFR with a significant increase in PFR during deterioration in our *in vivo* animal model and clinically, with a decrease in PFR observed during recovery of two patients whose lung function improved enough to be weaned from ECMO. The results imply that PFR can be used both for early detection of ARDS but also for monitoring the status of lung injury over time. With the help of PFR, the physicians may get quick and complementary clinical support to be able to assess lung function, which may reduce the need for invasive diagnostics and also help optimize or personalize therapies.

## CONFLICT OF INTEREST

All authors declare that they have no conflicts of interest.

## AUTHOR CONTRIBUTIONS

SL designed the study. MS, IS, SH, DB, AN, EG, PE, OH, LP, and SL acquired the data and performed data analysis. MS, IS, AN, DW, and SL prepared the manuscript. All authors have read and approved the final version.

## Supporting information



Supplementary Material
