# Supplementary material for: Monitoring lung injury with particle flow rate in LPS‐ and COVID‐19‐induced ARDS
Source: Physiol Rep. 2021 Jul 11;9(13):e14802. doi: 10.14814/phy2.14802 (PMC8273428; doi:10.14814/phy2.14802)
Supplement: Supplementary file 1 — Supplementary Material [file PHY2-9-e14802-s001.docx]

***Supplemental Methods***:

*Animal Preparation:*

Pigs were premedicated with xylazine (Rompun® vet. 20 mg/ml; Bayer AG, Leverkusen, Germany; 2 mg/kg) and ketamine (Ketaminol® vet. 100 mg/ml; Farmaceutici Gellini S.p.A., Aprilia, Italy; 20 mg/kg). Peripheral intravenous catheter (IV) in the earlobe, and urinary catheter in the bladder. Intubation was accomplished using a tracheostomy with a 7.5 size endotracheal tube. General anaesthesia with a ketamine (Ketaminol® vet), midazolam (Midazolam Panpharma®, Oslo, Norway) and fentanyl (Leptanal®, Lilly, France) infusion. A Siemens-Elema ventilator with non-humidified air (Servo 900C, Siemens, Solna, Sweden) was used for mechanical ventilation (MV). A pulmonary artery catheter with an introducer (Swan-Ganz CCOmbo V and Introflex, Edwards Lifesciences Services GmbH, Unterschleissheim, Germany) was placed in the right internal jugular vein and an arterial line (Secalon-T^TM^, Merit Medical Ireland Ltd, Galway, Ireland.) was placed in the right common carotid artery.

Lipopolysaccharide from Gram-negative bacteria *Escherichia coli* (O111:B4, Sigma-Aldrich, Merck KGaA, Darmstadt, Germany) was diluted into two solutions, one for endotracheal (ET) installation (0.33 mg/kg) and one for infusion in the pulmonary artery (2 µg/kg/min). Endotracheal installation of LPS (0.33 mg/kg) or saline was given as a single dose at time point zero. Arterial pulmonary infusion of LPS (2 µg/kg/min) or saline starts at time point zero and continues as an infusion (2 µg/kg/min) for one hour and was then reduced by 50% for another hour. Animals with hemodynamic instability (only observed in the LPS groups) required continuous infusion of norepinephrine (Pfizer AB, Sollentuna, Sweden) 40 µg/ml, 0.05 – 2 µg/kg/min and dobutamine (Hameln Pharma Plus gmbh, Hameln, Germany) 2 mg/ml, 2.5 – 5 µg/kg/min. Fluid loss was compensated by using Ringer’s acetate (Baxter Medical AB, Kista, Sweden) in all animals.

*Definition of ARDS*

Mild ARDS was defined as a PaO_2_/FiO_2_ ratio between 201-300 mmHg, moderate ARDS as between 101-200 mmHg, and severe ARDS as ≤ 100 mmHg. Pre-ARDS was defined as the time point between baseline and ARDS

In the patients, the cause of ARDS was established by testing. Three of the patients were COVID-19 positive in PCR testing of nasopharyngeal and bronchoalveolar lavage. One patient had a gram-negative bacterium induced ARDS.

*Hemodynamic measurements*

Heart rate (HR), systolic blood pressure (SBP), diastolic blood pressure (DBP), mean arterial pressure (MAP), central venous pressure (CVP), cardiac output (CO), systolic pulmonary pressure (SPP), diastolic pulmonary pressure (DPP), mean pulmonary pressure (MPP), pulmonary artery wedge pressure (Wedge), systemic vascular resistance (SVR), pulmonary vascular resistance (PVR) were all recorded.

*ECMO setup*

The ECMO equipment used was Medtronic Bio-Medicus® 560 centrifugal pump console, an Affinity® CP magnetic centrifugal blood pump and the TX50 Bio-Probe® Flow Transducer for measurement of the blood flow by conductivity. The circuit comprised of a microporous polypropylene hollow fiber oxygenator with a surface of 2.5 m^2^, Affinity Fusion™, a polyvinyl chloride 3/8-inch tubing set and a low prime centrifugal pump head AP40®. All components were coated with Cortiva® surface, an end point attached heparin surface (Medtronic Inc., , Minneapolis,USA). Heparin 1000 IU/ml was administered intravenously prior to cannulation and onset of ECMO and monitored with activated clotting time (ACT) targeting 180-220 sec. to avoid thrombotic events using a Hemochron Signature Elite® (Accriva Diagnostics Inc., San Diego, USA). Cannulation was performed with a 32Fr venous cannula was placed in the right atrium and the arterial cannula, 20 Fr, in the ascending aorta (DLP single stage venous cannula, EOPA Elongated One Piece Arterial cannula, Medtronic Inc., Minneapolis, USA).

V-A ECMO was chosen over V-V ECMO due to hemodynamic instability

*Measurements of cytokines using multiplex in plasma and bronchoalveolar lavage fluid (BALF)*

Plasma samples were collected in EDTA tubes and centrifuged before being stored at -80 degrees C. BALF was collected using 10 mL of saline wash.

*Measurements of proteins in EBP using o-link proteomics*

The analyses were performed using 1 μL of plasma for each panel of 92 proteins. Samples were quantified by real-time PCR using the Fluidigm BioMark™ HD real-time PCR platform (Briefly, for each protein a dedicated pair of oligonucleotide-labelled antibodies bind to the targeted protein and if the two oligonucleotides are in close proximity, a PCR target sequence is formed by a proximity-dependent DNA polymerization event and the resulting sequence is subsequently detected and quantified using real-time PCR. Each proximity extension assay (PEA) measurement has a specified lower detection limit (LOD) calculated based on negative controls that are included in each run and measurements below this limit were removed from further analysis. The Olink Multiplex data were reported in NPX (normalized protein expression levels), which are Ct values from the RT-qPCR reactions normalized by the subtraction of values for extension control, as well as an interplate control. The scale is then shifted using a runtime specific correction factor (normal background level). The final readout, NPX, is given on a log2-scale. Assay characteristics including detection limits and measurements of assay performance and validation for each protein are available at the manufacturer’s webpage (http://www.olink.com).

*Histology*

Biopsies were placed in 10% neutral buffered formalin solution (Sigma Aldrich, Germany) and left at 4°C overnight for fixation. Formalin-fixed tissues were subjected to graded ethanol series and isopropanol (both Fisher Scientific, UK) prior to paraffin embedding (Histolab, Sweden). 5 μm wide sections were cut and placed on microscope slides (Thermo Scientific, Germany) for staining. After de-paraffinization, the sections were stained with hematoxylin and eosin (Merck Millipore, Germany) followed by dehydration in consecutively graded ethanol and xylene solutions. Dried sections were mounted with Pertex (Histolab, Sweden).

The histological scoring was based on previously described main features of histological evidence of acute lung injury such as accumulation of inflammatory cells, formation of hyaline membranes, presence of protein debris in the alveolar space and thickening of the alveolar wall (1). Scoring was performed by 5 scientists on hematoxylin and eosin stained digital scans of slides; slides were randomized and blinded and all pigs were scored from all groups. Histological slides were scanned using Olympus VS120-S5 slide scanning system and blinded reviewers were supplied with digital scans extracted using OlyVIA at three different magnifications (8x, 40x, and 100x). For statistical analysis ANOVA - Dunnett's T3 multiple comparisons test was applied.

*Wet dry-weight ratio:*

Proximal lung tissue pieces harvested from the lower lobes in left and right lungs were weighed, lyophilized for 24 h, and then weighed again. The ratio between the wet and dry weight was then calculated. Two separate lung tissue pieces were analyzed from each location and time point.

References in Supplement:

1. Matute-Bello, Gustavo, et al. "An official American Thoracic Society workshop report: features and measurements of experimental acute lung injury in animals." American journal of respiratory cell and molecular biology. 44.5 (2011): 725-738.
